# Online Health Information Seeking by Parents for Their Children: Systematic Review and Agenda for Further Research

**DOI:** 10.2196/19985

**Published:** 2020-08-25

**Authors:** Christian Kubb, Heather M Foran

**Affiliations:** 1 Health Psychology Unit Institute of Psychology Universität Klagenfurt Klagenfurt Austria

**Keywords:** information seeking behavior, parents, child, internet, health behavior, digital health

## Abstract

**Background:**

Parents commonly use the internet to search for information about their child’s health-related symptoms and guide parental health-related decisions. Despite the impact of parental online health seeking on offline health behaviors, this area of research remains understudied. Previous literature has not adequately distinguished searched behaviors when searching for oneself or one`s child.

**Objective:**

The purpose of this review is to examine prevalences and associated variables of parent-child online health information seeking; investigate parents’ health-related online behavior regarding how they find, use, and evaluate information; and identify barriers and concerns that they experience during the search. Based on this analysis, we develop a conceptual model of potentially important variables of proxy online health information seeking, with a focus on building an agenda for further research.

**Methods:**

We conducted a comprehensive systematic literature review of the PsycINFO, JMIR, and PubMed electronic databases. Studies between January 1994 and June 2018 were considered. The conceptual model was developed using an inductive mixed methods approach based on the investigated variables in the study sample.

**Results:**

A total of 33 studies met the inclusion criteria. Findings suggest that parents worldwide are heavy online users of health-related information for their children across highly diverse circumstances. A total of 6 studies found high parental health anxiety, with prevalences ranging from 14% to 52%. Although parents reported wishing for more guidance from their pediatrician on how to find reliable information, they rarely discussed retrieved information from the web. The conceptual model of proxy online health information seeking includes 49 variables.

**Conclusions:**

This systematic review identifies important gaps regarding the influence of health-related information on parents’ health behavior and outcomes. Follow-up studies are required to offer parents guidance on how to use the web for health purposes in an effective way, as well as solutions to the multifaceted problems during or after online health information seeking for their child. The conceptual model with the number of studies in each model category listed highlights how previous studies have hardly considered relational variables between the parent and child. An agenda for future research is presented.

## Introduction

The proportion of individuals looking for health-related topics online has increased significantly in recent years [1]. Every second internet user in Europe has searched for health-related topics, such as diseases, injuries, or health promotion activities, at least once in the past 3 months [1]. Online health information–seeking (OHIS) behavior has been shown to affect the patient-doctor relationship [2], health care utilization [3,4], and multiple health outcomes [5].

However, large-scale studies suggest that about half of online health-related search sessions are not for one’s own health, but rather for someone else’s health situation [6,7]. These online health seekers are described in the literature under various terms [8-10] and there is no consensus about the definition of OHIS on behalf of others. The term “surrogate seekers” is sometimes used but has potential for confusion because “surrogate” is associated with surrogate pregnancies, surrogate motherhood, or sexual surrogates. The term is also misleading from an etymological point of view because it suggests that the search is a replacement or substitute for an action that would normally be done by the individual (Latin *surrogatus* means replace). This is particularly not the case in the parent-child search relationship.

We expand the definition on interactive health communication introduced by Robinson et al [11] by adding the term “proxy” seeking. The term “proxy OHIS” refers to any behavior of interactive health communication to obtain information in order to receive support or guidance on a health-related topic for someone else (eg, child, parent, grandparent, friend, neighbor, or any other relative or nonrelative).

Proxy searches are likely when there is a strong emotional tie between two people, which applies especially to intrafamily relations like parent-child relationships, intimate partner relationships, or other family relationships [7,10,12]. Parents consult the web widely for information about their child´s health symptoms and to assist in determining whether they need to seek medical aid for their child [13-15]. Therefore, information from the web can have a crucial impact on a child's health status, as parents use it to make health-related decisions by proxy.

The literature offers numerous studies on parental online information seeking related to their child’s health but so far, to our knowledge, there is only 1 literature review that attempts to summarize the findings. This integrative review by Park et al [13] included studies that do not differentiate between self-seeking and proxy seeking. Research has shown significant differences in characteristics of self-seekers and proxy seekers [7-9,16]. OHIS for oneself is typically based on different motives, needs, and circumstances than searching for someone else [8,16,17]. In addition, a recent study by Reifegerste and Bachl [18] suggests that it is not merely the individual factors in the seeker that have an influence on proxy seeking, but also relational factors between seeker and search subject and the relationship’s individual characteristics. For these reasons it is unclear whether reviews on the connection between OHIS and other outcomes, like patient-physician relationship [2], health anxiety [19], health literacy, or evaluation of online information [20] can explain the behavior of proxy seekers specifically. Other reviews have focused on general internet behavior of parents [21,22], maternal information-seeking behavior [23], or OHIS during pregnancy [24]; however, the parent-child search relationship was not examined exclusively.

Further, commonly used theoretical models only partially apply to understanding proxy-seeking behaviors. The comprehensive model of information seeking (CMIS) [25] is an established model to predict information-seeking behavior for individuals in different health contexts [26-28]. The influence of demographic variables, such as age, gender, or education, has been inconsistent in the literature on predicting proxy seeking [7-9,16]. Reifegerste and Bachl [18] concluded that further relational variables between searcher and search subject must also be considered in theoretical models to explain these differences. As another consequence, study results on prevalences and associated factors of proxy seeking are not readily transferable to parent-child proxy seeking, since the studies either did not specifically target parents but instead the general public [7-9], or they had a special search relation (eg, family caregivers to cancer survivors [16]).

For these reasons, this review specifically targets research on OHIS by parents. Online health seeking by parents for their children represents an understudied yet important area in the field of health internet research. The aims of this systematic review are (1) to examine prevalences and associated demographic variables of parent-child OHIS, (2) to investigate how parents find, evaluate, and use online health information, (3) to identify which barriers or concerns parents experience online, (4) to document important research gaps and formulate a research agenda, and (5) to develop a conceptual model on proxy OHIS.

## Methods

### Overview

This systematic review has been performed according to the Preferred Reporting Items for Systematic Reviews and Meta-Analyses (PRISMA) guidelines [29]. For a detailed description, see the PRISMA checklist in Multimedia Appendix 1.

### Data Sources and Search Strategy

A comprehensive analysis of the databases of PsycINFO and PubMed was performed. JMIR was also systematically searched. Starting from the word “surrogate seeking,” relevant core terms were identified and used for database analysis by applying the pearl finding and growing strategy [30]. These results were combined by using Boolean operators with family-related terms (mother, father, family, caregiver, parent, child). To consider the linguistic variations, these terms were truncated accordingly: (Mother* OR Father* OR Famil* OR Caregiver* OR Parent* OR Child*) AND (Internet OR Web OR Online OR Cyber* OR eHealth OR e-Health OR Health Information OR Information Seeking).

Studies from January 1994 to June 2018 were considered. The year 1994 was chosen because in this year, the first International World Wide Web Conference took place [31]. The existing web did not have essential health services at that time, and internet use was not common.

Records were summarized in a text-based database. After elimination of the duplicates, titles and relevant abstracts were reviewed. The full texts of the remaining records were reviewed to determine whether they met all inclusion criteria. A protocol of the process for selecting studies is available in Multimedia Appendix 2.

### Inclusion and Exclusion Criteria

To study OHIS by parents for their children, we included papers that met the following criteria: (1) the participants were parents; (2) the focus of the investigated behavior was OHIS on publicly available websites; (3) the online health-seeking behavior was for their own child; and (4) the study was written in English, presented quantitative data, and was published in a journal between 1994 and 2018.

First, the participants were parents. We defined parents as the primary caregivers who substantially support the child over a stable period in daily routines like feeding, hygiene, play, sleep, or health. Studies including other caregivers (eg, grandparents, other family members) besides parents were excluded if the percentage of other caregivers was greater than 5% of the total sample.

Second, the focus of the investigated behavior was OHIS on publicly accessible websites. Excluded papers included those about special online behaviors (evaluation of one specific website) or areas that are only accessible with registration (support groups, discussion boards, chats), papers that focused only on offline information-seeking behavior (books, television, physicians), and papers with a focus on non–health relevant search behavior.

Third, the online health-seeking behavior was for their own child. Studies with self-seeking behavior only (searching for own medical issues) and studies in which a self-seeking and proxy-seeking distinction was not made or was not possible (eg, pregnancy) were excluded.

Fourth, only papers written in English, presenting quantitative data, and published in a journal between 1994 and 2018 were included.

Based on studies that met the inclusion criteria, we manually reviewed their references to identify further studies that may not have been found through the literature review. Further, we used Google Scholar in June 2018 to identify cited papers that met the inclusion criteria.

### Selection of Studies

One author (CK) manually merged the studies from the different databases, removed duplicates, screened titles and abstracts for relevance, and hand-searched additional citations. The remaining records after screening by title and abstract were independently checked for eligibility by an author (CK) and a psychology master’s student (PS) (Cohen κ=0.84). In cases of nonagreement (7 out of 136), studies were discussed and a consensus for inclusion or exclusion was reached.

### Data Extraction and Analytical Strategy

The formal study characteristics were extracted from all 33 papers by 1 main author (CK) and can be found in their entirety in Multimedia Appendix 3 with a description of the studies (author, year of publication, location, survey period), study design (survey methodology, prospective vs nonprospective, cross-sectional vs longitudinal, hypothesis generating vs hypothesis testing, sampling technique), and sample characteristics (sample size, amount of parents in the sample, clinical vs community sample, parental gender, parental age, race, education, income, occupation, health insurance, child’s age). Subsequently, the content focus of each paper was coded in 3 category clusters: (1) studies with OHIS related to a child’s specific disease, (2) studies with OHIS related to a treatment or circumstance, and (3) studies that investigated parent-child OHIS in general.

We extracted the quantitative surveyed prevalences on parental OHIS as well as the related item that was used to assess prevalence because the study-specific prevalences are based on varying defined timespans. Significant and nonsignificant associated factors related to these items were extracted as well. To develop a research agenda, further information on theoretical frameworks, study limitations, and mentioned research gaps were extracted from the reviewed studies.

The heterogeneity of the sample composition of studies and the lack of a sufficient sample size of studies with similar outcome variables made the use of meta-analytical methods inappropriate for this review. Therefore, data were summarized by conducting a descriptive analysis and narrative synthesis. Frequency counts of key variables were coded and summarized.

### Coding for Conceptual Model

The conceptual model was developed with an inductive approach by the 2 authors (CK, HMF) based on the investigated variables in the studies. The CMIS by Johnson and Meischke [25] provides a theoretical framework and served as a basic structure to categorize the extracted variables. The underlying assumption of the CMIS is that characteristics of the individual and characteristics of the medium jointly influence health information–seeking behavior. Specifically, the model considers antecedents in the seeker (demographics, personal experience, beliefs, and salience), the characteristics and perceived utility of the information carrier (eg, health information on a website), and the final health information–seeking action (eg, decision to see a doctor). Based on the Johnson and Meischke [25] classification, we renamed the category names to make it more suitable for an internet search and distributed the constructs according to this distribution. Demographics, personal experience, beliefs, and salience of the CMIS are subsumed under “personal factors” and “environmental factors.” Characteristics and utilities of the CMIS are classified under “online search factors.” Information-seeking actions of the CMIS correspond to the outcome category labeled “health decision making and behaviors.” Finally, we have added the relational categories “relational factors” and “search subject” to our model. These are unique to health information seeking by proxy.

One author (CK) scanned the papers for quantified variables and created a binary coding system (1=variable is investigated; 0=variable is not investigated) with definitions for 49 variable categories to examine the frequencies of considered variables in the whole study sample (Multimedia Appendix 4). A psychology bachelor’s student (AS) and 1 author (CK) coded the variables in the studies independently (Cohen κ=0.69).

## Results

### Description of Studies

**Figure 1:**
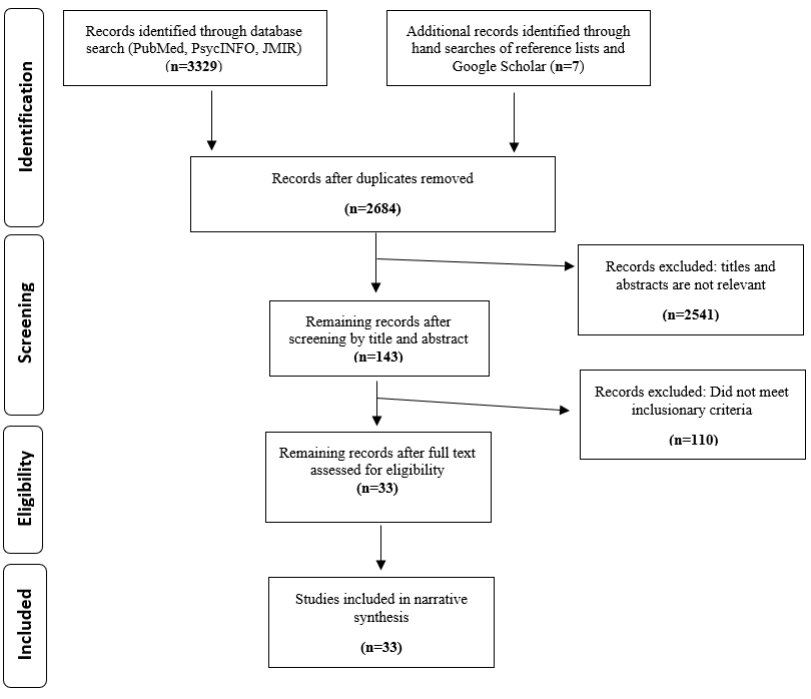
Flowchart of the systematic review search process. Adapted from Moher et al [32].

A total of 33 studies met the inclusion criteria (Figure 1 [32]). All studies were cross-sectional. Studies were conducted using in-person questionnaires (n=23), online surveys (n=5), interviews (n=2), telephone surveys (n=2), and mailed questionnaires (n=1).

The papers were sorted into 3 groups based on the focus of the paper (Table 1). A total of 13 of the studies focused on OHIS related to a specific disease or disease cluster, including asthma [34], attention-deficit/hyperactivity disorder [35], brachial plexus birth palsies [36], congenital heart disease [33,37,38], diabetes [39], hearing loss [40], hydrocephalus [41], scoliosis [42], skin disorders [43], and rare diseases [44,45]. An additional 13 studies addressed specific circumstances, mainly prior to a surgical procedure [46-51], after childbirth [52], 24 hours before an emergency department visit [53], during stay in a neonatal intensive care unit [54], in a pediatric outpatient clinic [55,56], in palliative care [57], and regarding attitudes towards human papillomavirus vaccination [58]. In addition, 7 studies dealt with general OHIS without specified diseases or particular circumstances [14,59-64].

**Table 1:** Study focus cluster.

Author	Year	Group	Specification
AlSaadi [34]	2012	Disease^a^	Asthma
Baker et al [42]	2012	Disease^a^	Scoliosis
Balkhi et al [39]	2015	Disease^a^	Diabetes
Ikemba et al [37]	2002	Disease^a^	Congenital heart disease
Kasparian et al [33]	2017	Disease^a^	Cogenital heart malformation
Lai and Mallory [43]	2000	Disease^a^	Skin disorders
Massin et al [38]	2006	Disease^a^	Congenital heart disease
Naftel et al [41]	2013	Disease^a^	Hydrocephalus
Nicholl et al [44]	2017	Disease^a^	Rare diseases
Porter and Edirippulige [40]	2007	Disease^a^	Deafness
Sage et al [35]	2017	Disease^a^	Attention-deficit/hyperactivity disorder
Shah et al [36]	2006	Disease^a^	Brachial plexus birth palsies
Tozzi et al [45]	2013	Disease^a^	Rare diseases
Boston et al [46]	2005	Circumstance^b^	Otolaryngology procedures
Dhillon et al [54]	2003	Circumstance^b^	Neonatal intensive care
Glynn et al [56]	2013	Circumstance^b^	Otolaryngology services
Hand et al [47]	2013	Circumstance^b^	Surgical procedure
Knapp et al [57]	2010	Circumstance^b^	Palliative care program
McRee et al [58]	2012	Circumstance^b^	Human papillomavirus vaccination
Nogueira et al [48]	2013	Circumstance^b^	Otolaryngology surgery
Semere et al [49]	2003	Circumstance^b^	Surgical procedure
Shroff et al [53]	2017	Circumstance^b^	24 hours before emergency department
Sim et al [50]	2007	Circumstance^b^	Surgical procedure
Slomian et al [52]	2017	Circumstance^b^	After childbirth
Tuffrey and Finlay [55]	2002	Circumstance^b^	Pediatric outpatients
Wong et al [51]	2017	Circumstance^b^	Surgical procedure
Harvey et al [59]	2017	General^c^	N/A^d^
Opeoluwa et al [60]	2017	General^c^	N/A
Pehora et al [61]	2015	General^c^	N/A
Sebelefsky et al [62]	2015	General^c^	N/A
Skranes et al [63]	2014	General^c^	N/A
Whyte and Hunter [64]	2008	General^c^	N/A
Yardi et al [14]	2018	General^c^	N/A

^a^“Disease” group indicates studies investigating parental online health information seeking related to specific illnesses, diseases, or disorders.

^b^“Circumstance” group indicates studies investigating parental online health information seeking related to a treatment or circumstance/situation.

^c^“General” group indicates studies investigating parental online health information seeking in general without a specified disease or circumstance.

^d^N/A: not applicable.

### Sample Characteristics

The samples from all studies included a total of 8665 participants and varied from a sample size of 70 [35] to 848 [58] participants, with a median of 209 participants (Table 2). A total of 26 out of 33 studies specified the proportion of mothers and fathers in the samples; with 4758 mothers and 1353 fathers, 77.86% were mothers (4758/6111). Of the 33 studies, 29 (88%) were conducted in the Western world, of which one-third of all studies (n=11) were conducted in the United States. Samples from other parts of the world included Nigeria [60], Singapore [51], Saudi Arabia [34], and Brazil [48].

Reported parental mean ages were all between 30 and 42 years, but only half of studies (n=16) reported ages of parent and child. The child’s age varied from neonates [37,54] to adults [40,42,44,45,55], but studies with reported mean ages or distributions consisted mainly of toddlers, preschoolers, and school-aged children aged 1 to 12 years. Adolescents were targeted in only one study explicitly [58]. Only 2 studies [34,62] differentiated between mothers’ and fathers’ demographic data and listed their information separately.

The samples consisted of highly educated parents, with more than 50% [33,34,36,41,44,48,54] and up to more than 75% of parents holding academic degrees [52,58,63], but 14 studies did not report any educational levels. The proportion of persons with only primary education varied between 0% [52] and 21.9% [57] among the studies that reported on education levels.

**Table 2:** Sample characteristics.

Author	Location	Sample size, n	Proportion of mothers, %	Sample	Child's age	Parent's age (years)
AlSaadi [34]	Saudi Arabia	500	—^a^	Clinical	<5 y (63.3%) >5 y (36.7%)	—
Baker et al [42]	Ireland	167	81	Clinical	Mean 11.9 y (SD 4)	<20 (12%) 20-35 (7%) 35-50 (75%) 50-65 (7%)
Balkhi et al [39]	United States	209	72	Clinical	Mean 12.26 y (SD 4.7)	Mean 42.15 (SD 8.94)
Boston et al [46]	United States	204	64	Clinical	—	Mean 34, range 16-65
Dhillon et al [54]	Canada	90	67	Clinical	2-148 days	Median 32
Glynn et al [56]	Ireland	501	75	Clinical	—	<18 (2%) 18-40 (68%) 41-65 (30%) >65 (<1%)
Hand et al [47]	Ireland	214	79	Clinical	—	<18 (1%) 18-40 (77%) 41-65 (21%)
Harvey et al [59]	Ireland	100	81	Clinical	<3 y (35%) 4-6 y (15%) 7-9 y (13%) 10-12 y (16%) >12 y (21%)	—
Ikemba et al [37]	United States	275	45	Clinical	Mean 4.3 y, range 7 d-24 y	—
Kasparian et al [33]	Australia	132	63	Clinical	Mean 21.8 months (SD 5.6)	Mean 35.2 (SD 7)
Knapp et al [57]	United States	129	90	Clinical	Mean 9.9 y (SD 6.1)	Mean 42.9 (SD 11.7)
Lai and Mallory [43]	United States	467	—	Clinical	—	—
Massin et al [38]	Belgium	389	47	Clinical	Mean 6 y (SD 4.9)	—
McRee et al [58]	United States	848	92	Nonclinical	First sample: Mean 14.7 y (SD 3.5); Second sample: Mean 13.9 y (SD 2.2)	First sample: <45 (28.2%) >45 (71.8%); Second sample: <45 (63.5%) <45 (36.5%)
Naftel et al [41]	United States	300	—	Clinical	Mean 8.2 y (SD 5.8)	Mean 36.7 (SD 10.4)
Nicholl et al [44]	Ireland	93	87	Clinical	<1 y (4%) 1-3 y (20.5%) 4-7 y (28.2%) 8-12 y (23.9%) 13-19 y (12.8%) 20-29 y (7.7%) 30-39 y (2.6%)	18-34 (24%) 35-49 (67%) 50-64 (10%)
Nogueira et al [48]	Brazil	132	83	Clinical	range 2-14 y	Mean 42, range 18-66
Opeoluwa et al [60]	Nigeria	142	100	Clinical	—	<20 (31.2%) 21-30 (42.2%) 31-40 (22%) >40 (4.6%)
Pehora et al [61]	Canada	146	—	Clinical	—	—
Porter and Edirippulige [40]	Australia	166	89	Clinical	<1 y (6%) 1-2 y (11%) 2-5 y (26%) 5-10 y (23%) 10-15 y (20%) 15-18 y (9%) 18-21 y (5%)	18-34 (29%) 34-49 (67%) 50-64 (4%)
Sage et al [35]	United States	70	81	Clinical	Mean 12 y (SD 2.6)	Mean 42.9 (SD 7.1)
Sebelefsky et al [62]	Austria	500	82	Clinical	Mean 2.4 y (SD 2.6)	Mean 34 (SD 6.4)
Semere et al [49]	United States	150	83	Clinical	—	Mean 35 (SD 11)
Shah et al [36]	United States	122	77	Clinical	—	—
Shroff et al [53]	United States	262	84	Clinical	Median 4 y (IQR 1.3-11)	Median 31 (IQR 25-37)
Sim et al [50]	United Kingdom	271	70	Clinical	—	—
Skranes et al [63]	Norway	99	100	Nonclinical	Mean 1.6 y, range 0.3-11 y	Mean 33.1, range 21-58
Slomian et al [52]	Belgium	349	100	Nonclinical	Mean 12.7 months (SD 14.5)	Mean 30.6 (SD 4.05)
Tozzi et al [45]	Italy	516	68	Clinical	Mean 10.3 y (SD 9)	Mean 42.7 (SD 9)
Tuffrey and Finlay [55]	United Kingdom	485	—	Clinical	Mean 6.3 y, range 4 weeks-23 y	—
Whyte and Hunter [64]	United Kingdom	245	—	Clinical	—	—
Wong et al [51]	Singapore	84	63	Clinical	Sample 1: median 5.1 y (range 0.2-15.7); Sample 2: median 9.8 y (range 0.6-15.9)	—
Yardi et al [14]	Australia	308	—	Clinical	—	<25 (7%) 25-44 (76%) 45-55 (15%) >55 (2%)
Total	N/A^b^	N=8665	77.86^c^	N/A	N/A	N/A

^a^Not available (exact numbers are not given by the author).

^b^N/A: not applicable.

^c^4758/6111. Calculated from the studies (n=26) that provided information on parental gender.

### Prevalence of Parental Online Health Information Seeking

Table 3 presents the prevalences of OHIS by proxy and associated factors, separated into general OHIS and OHIS for specific conditions in the child. In studies that reported prevalence by parental OHIS in general (n=9), prevalence ranged from 52% to 98%. Only 3 studies explicitly distinguished between general OHIS and specific OHIS [36,53,62]. Recent studies from 2017 or later showed the highest prevalences, with around three-fourths [51,59,60] to roughly 9 out of 10 parents who searched for health information related to their child [14,33,35,44]. Likewise, the health-related internet use among parents of children with rare diseases seems to be relatively high [44,45], even for older studies that deal with rare conditions [36,37]. Most of the data are related to OHIS before or because of surgical intervention [46-51]. In those cases, the prevalence varied between 38% and 90%.

In Table 3, all variables investigated in relation to parental OHIS are reported. Only a small proportion of studies provided bivariate or multivariate analysis of associated factors with OHIS, often presenting only selective data with significant outcomes. Education was shown to be the most common associated factor with parental OHIS [34,36,40,41,47,53,54,56,57], although some studies found no significant association with education [35,46,62].

The gender of the parent was not related to whether a parent searched the internet for their child in most studies [35,42,53,54,57,62], but it was related in one study [33]. The influence of the age of the parents on OHIS was inconsistent; some studies found younger age to be associated [56,62], one found older age to be associated [53], and other studies found no association with age and search behavior [14,35,40,42,47,57].

**Table 3:** Prevalence of online health information seeking and related factors.

Author	General OHIS^a^ for child^b,c^	Specific OHIS for child^b,c^	Associated factors	Nonassociated factors
AlSaadi [34]^d^	—^e^	79% (—/505) “Using the Internet to gain information on their children's [asthmatic] condition”	Father’s education, mother’s education, occupation of mother, nationality of father (Saudi vs non-Saudi), nationality of mother	Father’s nationality, occupation of father, history of allergy
Baker et al [42]^d^	—	58% (97/165) “Have you searched the internet for information on scoliosis?”	Corrective surgery, private health insurance	Postoperative complications, parent gender, education, child age, parent age group, visit type, home internet access
Balkhi et al [39]^d^	—	64% (133/209) “Using the Internet for diabetes information”	Child‘s age	HbA_1C_^f^ level
Ikemba et al [37]^d^	—	58% (93/160) “Used the Internet to obtain information related to their child's cardiac diagnosis”	—	Type of congenital heart defect
Kasparian et al [33]^d^	—	91% (—) “Identified the internet as a source of congenital heart disease information”	Parents' gender (mothers)	—
Lai and Mallory [43]^d^	—	13% (62/467) “Used the Internet to search for information related to their child's skin disorders”	—	—
Massin et al [38]^d^	—	35% (84/238) “Used the Internet to obtain information related to their child's cardiac diagnosis”	Expected treatment modalities	Type of congenital heart defect, internet access at home
Naftel et al [41]^d^	—	82% (225/275) “Searching for hydrocephalus-related information online”	Caucasian, income, education	Geographic location (urban vs rural), parents' age, etiology of hydrocephalus
Nicholl et al [44]^d^	—	92% (105/114) “Use the Internet to find information about your child's condition [at least every few month]”	—	—
Porter and Edirippulige [40]^d^	—	82% (131/159) “Use the Internet to ﬁnd information about deafness and related topics [at least every few months]”	Education	Parents' age, child’s age, geographic area, employment status, type of hearing loss
Sage et al [35]^d^	—	87% (61/70) “Searching the Internet for ADHD[^g^] information”	—	Parents' age, parents' gender, years of education
Shah et al [36]^d^	90% (—/122) “Searched the Internet for health-related information at least once a month”	88% (108/122) “Used the Internet to search for information on Brachial Plexus Birth Palsies”	Education, income	—
Tozzi et al [45]^d^	—	99% (462/468) “Information searched on the web [related to disease characteristics]”	—	—
Boston et al [46]^h^	—	49% (83/170) “Used the Internet to look for information about their child's diagnosis and/or surgical [otolaryngology] procedure”	—	Education, frequency of internet use
Dhillon et al [54]^h^	—	44% (40/90) “Having searched the Internet for information related to the medical condition of their baby [in the neonatal intensive care unit]”	Education, parents' age	Parents' gender, employment status, comfort in English
Glynn et al [56]^h^	—	30% (149/497) “Had searched online for information regarding their child's ENT[^i^] problem”	Education, parents' age, private health insurance, daily internet use, smartphone	—
Hand et al [47]^h^	—	38% (82/214) “Searched the internet regarding their child's surgical issue”	Education, private health insurance, daily internet use, smartphone	Parents' age
Knapp et al [57]^h^	—	81% (92/114) “Used Internet information about their children's health [who have life-threatening illnesses]”	Education, parents' race, language spoken at home (English)	Parents' gender, parents' age, marital status, type of household, sibling in household, children's age, children's health
McRee et al [58]^h^	—	21% (154/773) “[Mothers] having heard about HPV[^j^] vaccine through the Internet” 17% (19/115) “[Fathers] having heard about HPV vaccine through the Internet”	Greater knowledge about HPV	—
Nogueira et al [48]^h^	—	90% (117/130) “Look[ed] for information on the Web on the condition of your child/guardian [with undergoing otolaryngology surgical procedure]”	—	—
Semere et al [49]^h^	—	69% (88/128) “Searched for information relating to their child's surgery procedure or treatment”	—	—
Shroff et al [53]^h^	52% (117/224) “At least one episode of Internet use for general pediatric health information in the preceding 3 months”	12% (31/262) “Used Internet in 24 hours prior to emergency department visit”	Education, income, older children, older parents	Parents' gender, race of parent, race of child, insurance, triage classification, time of enrollment, disposition
Sim et al [50]^h^	—	53% (144/271) “Had accessed the Internet to seek more information regarding their children's condition [surgical outpatient]”	—	—
Slomian et al [52]^h^	—	12% (43/349) “Seeking information for the baby only [after childbirth]” 75% (262/349) “Seeking information about themselves or about their baby [after childbirth]”	—	—
Tuffrey and Finlay [55]^h^	—	22% (107/485) “Used the internet to ﬁnd information about the problem for which they were being seen in clinic that day”	Internet access at home	—
Wong et al [51]^h^	—	74% (62/84) “Use the Internet to access child's current condition [surgical procedure]”	—	—
Harvey et al [59]^k^	72% (72/100) “Frequency of use of the Internet to access healthcare information at least yearly or less”	—	—	Children with chronic diseases
Opeoluwa et al [60]^k^	77% (109/142) “Had ever consulted the Internet to find answers to their babies' medical problems or health-related issues”	—	Self-medication, health-seeking behaviors	—
Pehora et al [61]^k^	98% (143/146) “Using the Internet to search for health information regarding their child [at least few times a year]”	—	—	—
Sebelefsky et al [62]^k^	94% (471/500) “General internet use to obtain child health information [at least occasionally]”	21% (105/499) “Internet use to be informed about the reason for consultation [pediatric outpatient clinic]”	Parents' age (younger parents), younger children	Parents' gender, nationality, education, children's sex, children's diet
Skranes et al [63]^k^	98% (97/99) “Used the Internet regularly to search for child health information”	—	—	—
Whyte and Hunter [64]^k^	64% (121/190) “Used Internet to search for information regarding child's health”	—	—	Scottish Index of Multiple Deprivation
Yardi et al [14]^k^	90% (276/308) “Searching for medical information about their child's health”	—	—	Parents' age, number of children, inpatient/outpatient, parent-perceived seriousness of child's condition

^a^OHIS: online health information seeking.

^b^Percentages are rounded.

^c^Textual information in brackets has been added for better understanding.

^d^Group 1: study investigated parental OHIS related to specific illnesses, diseases, or disorders.

^e^Not available (exact numbers or information not given by the author).

^f^HbA_1C_: glycated hemoglobin.

^g^ADHD: attention-deficit/hyperactivity disorder.

^h^Group 2: study investigated parental OHIS related to a specific treatment or circumstance/situation.

^i^ENT: ear, nose, and throat.

^j^HPV: human papillomavirus.

^k^Group 3: study investigated parental OHIS in general.

### How Parents Find, Evaluate, and Use Health Information

Google was reported to be the most common starting point for gathering health information [33,42,44,48,50,51,53,62,63]. The most recent studies found that 9 out of 10 parents use Google [33,44,51] and many of the daily internet users go online via their mobile phones [45]. The rising trend in mobile phone use over desktop computer use was already evident in the studies since 2013 [41,44,45,47,52,53,56]. There is some evidence that smartphone owners are more likely to look for health-related information relating to their child [47,56] than people without a smartphone. Yardi et al [14] reported first that smartphones have overtaken the desktop computer as the most used device for proxy health information seeking.

Parents described information from the web mostly as helpful and useful, with a fundamentally positive attitude towards OHIS [33,34,37,38,41-52,56,57,63,64]. The most frequent underlying search motive was the need for a better understanding of the child's condition, which gave parents the opportunity to play a more active role in the management of their child's health [14,33,34,42,43,45,49,51,52,55].

Parents used the internet to decide if their child needed a doctor [14,60,63] and in some cases also in emergency situations [53]. Likewise, they searched the web before a doctor’s visit to prepare for the appointment and after the doctor’s visit to address unanswered questions [14,33,42]. The web was also reported to be used as a second medical opinion, but the amount varied from 1% to 57% across studies covering different medical circumstances [44,45,51,52].

Unfortunately, only a few studies examined search content in detail. Information about characteristics of specific diseases, current treatments, and diagnoses were the most common search topics [36,44,45,50,51,55,59], while looking for alternative treatments [40,44,50,51,55] was comparatively less common. However, the choice of treatment could be influenced by the information from the internet [46-48,56]. Studies that did not restrict their items on search content to a specific disease showed a greater variety of search content, including searches for health purposes like children’s nutrition [44,45,52,61] or development [44,52,61].

A consistent finding across studies over time was the search for or use of support groups [14,33,39-41,44,45,49-51,55,57]. In particular, parents of children with chronic, acute, or rare diseases showed a high need for support groups [40,41,44,45,57].

### Barriers and Concerns That Parents Experience Online

Parents perceived the information on the web as easy to understand [14,43,46-48], but studies found that parents sometimes had problems distinguishing between trusted and untrusted websites [14,33] or finding reliable information [54,60]. Some studies showed only a small proportion of parents who considered the reliability and trustworthiness of the information [14,49,51], while other studies showed greater skepticism of the participants towards the internet as a reliable source [41,54]. Further, parents did not necessarily navigate to the pages that they trusted or that provided trustworthy information [61]. The web as a trusted source was ranked lowest, but it is used frequently as a source of health information [46,54], and even unreliable information was reported as helpful [52,54].

Although parents wished for more guidance regarding good websites from their physicians [14,41,48,52,53], parents rarely or never discussed their findings with them [14,34,40,46-48,50,51]. Reasons for not discussing findings included a lack of time and a fear of doctors’ disapproval [33,51,52,59]. Other problems mentioned included nonnative language information [34,38], technical language [34,51], and information overload [33,38,51].

There were 6 studies that reported anxiety, distress, or worries caused by information from the internet [33,42,44,45,50,51]. The proportion of affected parents was between 14% [51] and 52% [45]. Nicholl et al [44] reported that online searching lowered anxiety in 16% of their participants, but the number of people with increased fear was twice as large. Likewise, attention should also be paid to parents who spend a lot of time searching the web for health purposes or who visit many different sites, like in Shah et al [36] or Porter and Edirippulige [40], which indicates that some parents may not be able to find the health information they need.

### Model of Online Health Information Seeking by Proxy

**Figure 2:**
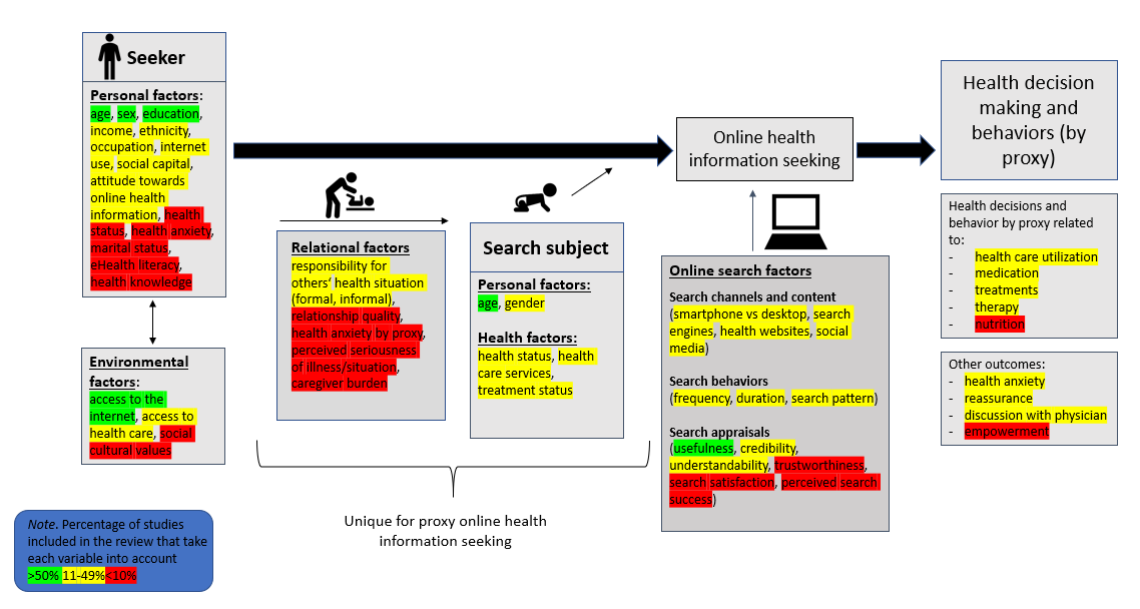
Model of proxy online health information seeking and decision making.

The conceptual model (Figure 2) consists of 6 categories: personal factors within the seeker, environmental factors, relational factors between seeker and search subject, factors within the search subject (ie, the child in regard to personal and health variables), online search factors (search channels, content, behaviors, and appraisals), and outcomes. Factors of the search subject and the relational factors are unique for OHIS by proxy.

The most commonly studied variables were the age of the parent (21/33), their gender (23/33), their education (21/33), and their access to the internet (25/33), as well as the age of the child (21/33). Attitude on usefulness of online health information was the only variable from the other categories that was frequently included in studies (22/33). The most neglected study variables were relational variables. However, variables from the category of online search factors were also rarely assessed, especially trustworthiness of information (2/33), search satisfaction during the search (2/33), and the perceived search success after the search (1/33). In addition, influencing factors such as trait health anxiety (0/33), health knowledge (2/33), and eHealth literacy (2/33) were also considered in less than 10% of the studies.

The surveyed variables of the studies that met the inclusion criteria for narrative synthesis (N=33) were counted. On average, studies considered 12.12 variables (SD 5.36) out of 49 coded variables (Multimedia Appendix 4). The study by Whyte and Hunter [64] presented the fewest number of variables (n=2) and Kasparian et al [33] considered the most variables (n=28).

## Discussion

### Principal Findings

Parents are heavy users of health-related information on the internet for their children across highly diverse circumstances. Across studies, results showed that the majority of parents have searched the web at least once for general health information for their child. This indicates that the percentage of parents who search for their child is well above the national averages for self-seekers [1]. Education was the most consistent predictor for proxy OHIS across all studies. Well-educated parents used the internet for their children more than parents with little education. The most recent studies show that Google search engine was used by almost all parents as a starting point for OHIS.

Further, information retrieved from the web was reported to be used by caregivers for decision making about children’s health. Physicians should be aware that parents reported using information found on the web under certain circumstances for treatment choice or to make health care utilization decisions. Although parents rarely or never discussed information from the web with their doctor, studies showed that they would like more support from doctors on how to find reliable sources. However, there is a tremendous lack of understanding about which criteria parents use to make decisions and about individual and external factors that contribute to parental empowerment. More research is needed on offering parents tangible knowledge and appropriate guidance, using the web for health purposes in an effective way, and problem solving approaches to the multifaceted problems that come up during or after OHIS for their child (eg, unmet information needs, obstacles in parent-doctor communication, false proxy lay diagnoses by parents with wrong conclusions for child’s needed treatment, unnecessary or missed doctor visits, and parental health anxiety due to online health information). All 6 studies that surveyed anxiety and distress by proxy showed a significant proportion of affected parents.

### Implications for Future Research

Overall, this review identified the need for more developed research in the area of OHIS. As can be seen from the results of the review, most studies provided descriptive information, but process- and theory-driven advances in this research area are still in infancy. To facilitate more systematic research in the area of OHIS, we present a summary of research gaps in the context of the conceptual framework provided. A total of 17 studies included in this review named research gaps explicitly or gave suggestions for further research. We synthesized these into the results of the developed model on proxy OHIS and the current research on proxy health searches. This is a framework that can be used for future studies.

#### Differentiation of Self-Seekers and Proxy Seekers

First, we recommend a clear separation between parental self-seeking and parental proxy seeking. These health behaviors represent two independent processes with different motivations, circumstances, and predictors [7-9,16,17]. If both are considered in one study, authors must state explicitly which they are referring to. Numerous excluded studies mixed them or formulated the research items in a vague or undifferentiated way. Furthermore, it is largely unknown whether findings from parent-child OHIS also apply to other types of proxy seeking, such as searching for a spouse or parents. For instance, existing research indicated that proxy seekers tend to be women [7,16], but this review showed that the gender of the parent had no influence on whether they searched the internet for their child. As suggested by Reifegerste et al [17], relational factors are relevant variables for proxy seeking and therefore a fundamental part of our conceptual model. They could explain contradictory results from past research. Relational variables like relationship closeness and quality should be considered in future studies.

#### Representative Samples and Generalizability

Second, studies with generalizable samples are urgently needed to provide an accurate estimate of the actual prevalence and influencing factors of parental proxy seeking. The lack of generalizability of the results is the most frequently mentioned limitation, which leads to the recommendation for larger and more diverse samples in further studies [33-35,51,54,57,61,63]. None of the studies in this systematic review had a representative sample. Existing literature mainly offered convenience samples in clinical environments with specific populations of ill children. However, it is unclear whether the results are also generalizable to parents of children who are not seriously ill and whether general patterns across proxy seekers can be established. In addition, systematic studies from non-Western countries have so far been lacking, for example in Asian and African regions, where smartphone and internet use has increased substantially in recent years [65]. There is virtually nothing known about how parents search and behave in low-income countries, where they have access to the web but may have limited access to some health care options.

#### Theoretical Frameworks

Third, the theoretical approaches are still lacking after 20 years of research in the field of parental OHIS. Only 3 studies [57,58,60] referred to existing theories at all, and none of these studies used them to interpret their results. Existing theory-based literature on models of OHIS did not consider proxy seekers [66]. Nonetheless, health characteristics of the supported search participants are associated with the search behavior of the proxy seeker [17,67]. To address this gap, we present a conceptual model on OHIS by proxy. This framework can be used for future studies in order to consider important influencing variables on parent-child OHIS.

#### Advanced Modeling Techniques

Fourth, data analyses in previous studies have been limited in scope. New studies should analyze the collected data with advanced statistical methods and go beyond the solely descriptive approach that has commonly been used so far. Structural equation modeling could be beneficial for testing the conceptual model proposed. Further, longitudinal analyses would help explore search behaviors and their connection with health care decisions and health care utilization behaviors over time.

Dyadic data analysis could be used to test both parents’ search behaviors in the context of the conceptual model. Results of this review found that both mothers and fathers searched for health-related information regarding their children [35,42,53,54,62]. To what extent they differ in search behaviors and whether interpersonal interactions influence search behaviors could be examined in future dyadic studies. Literature suggests that fathers’ involvement can impact a child’s social, behavioral, and psychological outcomes [68], and the results of this review reveal that social capital is an important variable that has been included in approximately half of the studies. Dyadic modeling could help address the question of how co-occurring proxy seeking by mother and father affects their health decisions and their child’s health outcomes.

#### Social Media

Fifth, upcoming studies need to focus more on the new possibilities on the internet. The landscape for consuming health-related information is completely different than it was ten years ago but it is hardly studied for parental proxy seeking. Facebook, Twitter, and YouTube are heavily frequented to find and share health information, but parental social media behaviors are not well understood. In particular, trustworthiness of information online was found in our review to be understudied, with only 2 studies examining this construct. This may be more important than ever due to the challenges related to health misinformation and fake news on social media [69,70]. In this context, the impact of far-reaching influencer personalities on platforms such as Instagram on the health behavior of young parents has barely been considered in the literature on OHIS. So far, it is also unknown whether the use of smartphones instead of desktop computers has fundamentally changed the search for health-related information, since these devices are now accessible immediately and everywhere.

#### Factors of a Successful Online Health Search and Interventions to Improve Search Skills

Sixth, evidence on factors that result in search success among parents searching for health information is lacking. Mixed method approaches with eye tracking, desktop tracking, or think-aloud protocols with evaluation immediately after a health-related search could contribute to better understanding of which parental factors (eg, eHealth literacy) and search process factors (eg, number and choice of sources, search duration) might be associated with a positive search outcome that empowers parents. Based on those types of studies, evidence-based recommendations for parents could be formulated for use on health-related sites on the internet. The online search factors category of the conceptual model (Figure 2) presents nonpersonal related variables that may play a role in the search process only. In addition, to move forward in the research area of search success, new psychometrically tested measures that operationalize search success in a valid and reliable way will need to be developed.

The question of how to improve parental searching skills with interventions is also in need of further research [14,58,63]. It is unclear how parents can be empowered effectively for OHIS [14] and if educational interventions are able to improve parents’ health information–seeking skills on a long-term basis [33]. Research on approaches and skills to teach parents appropriate and effective methods of proxy OHIS are still needed [14,33,40].

#### Suffering From Online Health Information Seeking

Seventh, the negative accompaniments of OHIS, such as uncertainty, anxiety, or triggered health care utilization, are well described among self-seekers [4,71-73] but rarely investigated for proxy seekers [74]. Some studies have documented that parents are also negatively impacted from information seeking [33,42,44,45,50,51]. However, there is a lack of research that applies approaches to improve the outcomes for parents who currently do not benefit from proxy OHIS. Moreover, taking into consideration the relational aspect between seeker and subject may lead to a better understanding of the prevention of negative outcomes for parents searching for health information [75].

#### Effects on the Doctor-Parent Relationship

Eighth, the role of health professionals and their reciprocal communication with online health–seeking parents needs more investigation. Research gaps concern doctors’ perceptions of eHealth resources [33], their responses to parents’ retrieved online information [44], and the effects of doctor engagement in the doctor-parent relationship [33]. Searching for health information on the internet can have a positive effect on the doctor-patient relationship among self-seekers [76]. Future studies will need to examine if and under what circumstances this applies to proxy seekers. Subsequently, more research is needed on how pediatricians can support parents in their OHIS behavior (eg, with a proactive conversational approach during appointments or evidence-based leaflets with instructions and links to reputable websites). Unfortunately, studies on doctor-parent communication improvements related to OHIS by proxy or on standardized information leaflets are lacking.

### Limitations

This systematic review has several limitations. We included studies from a period of 17 years, while the manner of OHIS has undoubtedly changed much faster. The circumstances in which the studies were conducted may be difficult to compare due to differences at the point of data collection, geographical location, characteristics of the parents, and the underlying diseases of the children. Further, almost all studies were conducted in clinical settings, and the findings in this review may not generalize to other populations. There is a strong need for research on representative samples of parents. Estimates of the prevalence of proxy OHIS should be treated with caution, as it was often not consistently defined in the previous studies, with different time periods being queried and the health status of the child varying.

### Conclusions

Our systematic review has important implications for future research. The results suggest that more studies on parental OHIS are needed to understand parental online search behaviors and support parents in their medical decision making by proxy. There is evidence that parental proxy OHIS is a very common but understudied behavior. Our presented agenda has highlighted research gaps that will hopefully lead to more systematic, theoretically informed research in this field.

## Appendix

Multimedia Appendix 1 PRISMA checklist.

Multimedia Appendix 2 Protocol of the process for selecting studies.

Multimedia Appendix 3 Study and sample characteristics.

Multimedia Appendix 4 Coding scheme for model of proxy online health information seeking and decision making.

## Abbreviations

CMIS: comprehensive model of information seeking
OHIS: online health information seeking
PRISMA: Preferred Reporting Items for Systematic Reviews and Meta-Analyses
